# Consensus document on the role of cardiac computed tomography for pre-procedural planning in minimally invasive percutaneous coronary intervention from the Japanese association of cardiovascular intervention and therapeutics

**DOI:** 10.1007/s12928-025-01234-2

**Published:** 2026-01-03

**Authors:** Kenji Sadamatsu, Fuminobu Yoshimachi, Naoki Masuda, Shinichiro Yamada, Tomokazu Ikemoto, Nozomi Kotoku, Munenori Okubo, Yoshio Kobayashi, Ken Kozuma

**Affiliations:** 1 Department of Cardiology, Omuta City Hospital, 2-19-1 Takarazaka-machi, Omuta, 836-8567 Fukuoka Japan; 2 Division of Cardiology, Department of Internal Medicine, Tokai University Hachioji Hospital, Hachioji, Japan; 3 Department of Cardiology, Shonan Oiso Hospital, Oiso, Japan; 4 Division of Cardiology, Kita-Harima Medical Center, Ono, Japan; 5 Department of Cardiology, Japanese Red Cross Kumamoto Hospital, Kumamoto, Japan; 6 Division of Cardiology, Department of Internal Medicine, St. Marianna University School of Medicine, Kawasaki, Japan; 7 Department of Cardiovascular Medicine, Gifu Heart Center, Gifu, Japan; 8 Department of Cardiovascular Medicine, Chiba University Graduate School of Medicine, Chiba, Japan; 9 Department of Cardiology, Teikyo University Hospital, Tokyo, Japan

## Abstract

Slender percutaneous coronary intervention (PCI) is a minimally invasive technique that uses smaller catheters, typically 5-French devices, to reduce bleeding complications and eliminate the need for unnecessarily large catheters. While these techniques are highly effective for non-complex lesions, they face inherent challenges, such as limitations in device compatibility and technical constraints. These challenges emphasize the importance of thorough pre-procedural planning to ensure optimal equipment selection and successful procedures. Cardiac computed tomography (CT) addresses these limitations by providing a comprehensive three-dimensional view of the coronary artery anatomy and lesion characteristics. This consensus statement outlines the role of cardiac CT in optimizing slender PCI strategies, emphasizing the importance of detailed anatomical assessments and advanced evaluations of lesion complexity. Cardiac CT enables precise measurements of vessel dimensions, identification of optimal landing zones, and accurate characterization of lesion complexity, including calcified plaque. Thin-slab maximum intensity projection reconstruction provides simultaneous longitudinal and cross-sectional views, comparable with findings of coronary angiography and intravascular imaging, facilitating interventional planning. For complex lesions, CT is essential. It accurately predicts the risk of side branch occlusion in bifurcation lesions, characterizes the distribution of calcification in heavily calcified lesions to aid in device selection, and helps in the appropriate selection of cases for chronic total occlusion lesions based on CT-derived scores and detailed morphological assessments. To systematically apply these principles, a practical pre-procedural checklist for CT-guided planning is proposed. The integration of cardiac CT into the slender PCI workflow extends beyond anatomical assessment to encompass the optimization of resource utilization, potentially enabling interventionists to proceed with appropriately minimally invasive techniques from the outset.

## Introduction

This consensus statement, prepared and discussed by an expert panel of the Japanese Association of Cardiovascular Intervention and Therapeutics and the Slender Club Japan, provides a comprehensive overview and recommendations on the critical role of cardiac computed tomography (CT) for pre-procedural planning in slender percutaneous coronary intervention (PCI).

Transradial coronary intervention has emerged as the preferred vascular access route for PCI, offering significant reductions in bleeding complications and improved patient outcomes compared with transfemoral access. However, the conventional use of 6-French (6-Fr) catheters with an outer diameter of 2.7 mm often exceeds the diameter of radial arteries, particularly in patients with small vessel caliber, leading to procedural difficulties, patient discomfort, and increased risk of radial artery occlusion [1, 2]. Thus, further downsizing of catheters, typically 5-Fr devices, should be the next step to reduce bleeding complications and radial artery occlusions [3–5]. Similar to catheter downsizing, reducing contrast medium use and radiation exposure, shortening procedure time, and adopting simple yet effective strategies have all converged into the concept of minimally invasive catheter-based treatment termed slender PCI [6–8]. The concept has since evolved, encompassing various innovative strategies that include miniaturization of materials, sheathless coronary intervention [9, 10], and specialized backup support techniques [11]. These strategies collectively enable complex coronary interventions through smaller access sites while maintaining procedural efficacy and safety. Collaborative efforts stemming from this philosophy led to the establishment of the Slender Club Japan and Europe [6].

The purpose of slender PCI is to achieve efficient treatment with the minimal necessary system, thereby reducing the risk of complications. This beneficial effect has been verified in analyses of the Japanese Association of Cardiovascular Intervention and Therapeutics (CVIT) registry data [8]. However, collection and careful analysis of sufficient pre-procedural information is essential to establish a reliable procedural strategy for performing slender PCI safely and effectively. Therefore, the successful implementation of slender PCI requires meticulous pre-procedural planning to optimize equipment selection and predict procedural feasibility. While slender PCI can effectively manage relatively non-complex lesions with a high degree of safety and efficacy, downsizing the catheter has inherent disadvantages, including limitations in device compatibility and various technical constraints (Table 1). These limitations pose significant challenges in more complex cases, particularly for operators unfamiliar with the technique.

Traditional angiographic assessment provides limited information regarding coronary and other arterial characteristics, including dimensions, tortuosity, and anatomical variants that critically influence the choice between conventional and slender PCI approaches. Moreover, slender PCI systems face challenges in accommodating multiple device insertions and providing adequate backup support. These factors make comprehensive lesion assessment and procedural strategy planning essential for successful outcomes. Insufficient pre-procedural evaluation can result in equipment mismatch and inadequate backup support, leading to longer procedure times, increased radiation exposure, and conversion to systems with larger catheters or alternative access routes.

Cardiac CT has emerged as a crucial imaging modality that addresses the limitations of conventional angiographic planning for slender PCI procedures. Comprehensive three-dimensional (3D) visualization of both coronary arteries and access route anatomy by cardiac CT enables precise measurement of vessel diameters, characteristics, assessment of anatomical variants, and evaluation of access vessel suitability for slender PCI [12, 13]. The non-invasive nature of cardiac CT allows for detailed pre-procedural evaluation without additional procedural risks, facilitating optimal equipment selection and development of the procedural strategy. Importantly, cardiac CT optimizes resource utilization by accurately determining the appropriate level of procedural complexity. This allows for the identification of cases requiring advanced techniques and the confident exclusion of complex anatomical features in cases suitable for slender approaches. This “rule-out” capability enables interventionists to proceed with appropriately minimally invasive techniques from the outset, avoiding unnecessary equipment upgrades and enhancing procedural efficiency. While the role of CT guidance in conventional PCI remains under investigation with limited definitive evidence, the unique constraints of slender PCI create a compelling rationale for enhanced pre-procedural planning. The limited device options, inability to accommodate multiple device insertions, and potential challenges with backup support in slender PCI systems amplify the importance of accurate lesion assessment and optimization of the procedural strategy. This comprehensive imaging capability establishes cardiac CT as an indispensable tool for enhancing the safety, efficacy, and success rates of slender PCI procedures.

Given these advantages of cardiac CT-guided planning, the broader adoption of Slender PCI becomes increasingly feasible. Slender PCI is often regarded as a minimally invasive technique practiced by a limited number of operators. Nevertheless, analyses of the CVIT registry data have suggested that, in elective procedures, slender PCI demonstrates superiority over conventional approaches in all complications except in-hospital mortality [8]. Therefore, this document has been prepared and published as one of the key resources to promote the wider implementation of slender PCI among members of the CVIT and internationally.

### Cardiac CT acquisition and reconstruction technique

Modern cardiac CT protocols for PCI planning must optimize both temporal and spatial resolution, while minimizing the dose of radiation and use of contrast medium. High-resolution coronary CT angiography, with a slice thickness of 0.5–0.75 mm and temporal resolution ≤ 100 ms, is typically performed using prospective electrocardiography-triggering (ECG-triggering) or retrospective ECG-gating techniques to minimize motion artifacts. Optimal image quality necessitates heart rate control through beta-blockade (target: <60 bpm) [14, 15], and nitroglycerin administration for coronary vasodilation. Considering the minimally invasive nature of slender PCI procedures, acquisition protocols strive for dose optimization where feasible, while ensuring diagnostic image quality for pre-procedural planning. Strategies currently being considered or actively explored for this purpose include reducing tube voltage (e.g., 80–100 kVp), modulating tube current, and shortening scan ranges. Contrast medium volumes are minimized to 40–60 mL through optimized injection protocols and personalized timing. Image reconstruction uses advanced iterative algorithms and deep learning techniques to enhance diagnostic image quality, while minimizing radiation exposure.

**Fig. 1:**
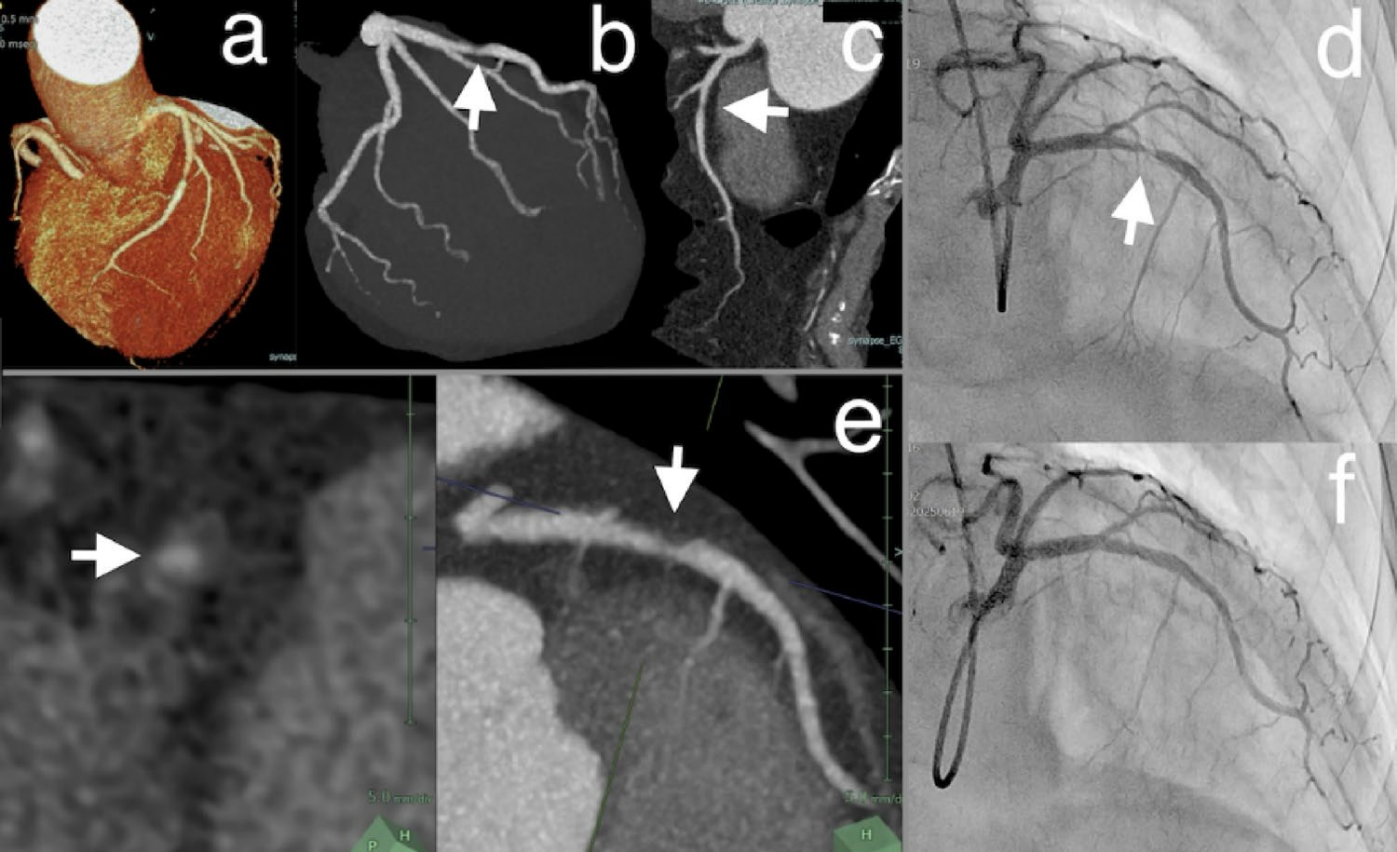
Cardiac CT and coronary angiography findings in a patient with a left anterior descending artery lesion. (**a**) A volume-rendered reconstruction of the coronary arteries demonstrates the overall anatomy. (**b**) A full-volume maximal intensity projection reveals a stenotic lesion in the left anterior descending artery. (**c**) A curved multiplanar reformation provides a detailed view of the lesion characteristics, though its 2D projection does not offer an accurate representation of the original 3D morphology. (**d**) Coronary angiography confirms severe stenosis in the left anterior descending artery. (**e**) Thin-slab MIP demonstrates a similar image to the coronary angiography, and the short-axial view reveals an eccentric low-density plaque. (**f**) Post-intervention coronary angiography shows successful placement of a drug-eluting stent using a slender 5-Fr guiding catheter. Arrows indicate the stenotic lesion. *2D* two-dimensional, *3D* three-dimensional, *CT* computed tomography, *Fr* French, *MIP* maximum intensity projection

Thin-slab maximum intensity projection (MIP) is a crucial post-processing technique that enhances the visualization of coronary artery anatomy for interventional planning [16]. Standard CT analysis typically employs several image reconstructions, including full-volume maximal intensity projections, standard axial views, multiplanar reformations (curved and straight), and short-axis cross-sectional images (Fig. 1). Thin-slab MIP typically creates slab projections with a thickness of 5 mm, consolidating multiple thin slices into a single image. Additionally, the cross-sectional view can be displayed side by side. Thus, this technique offers simultaneous longitudinal views, akin to coronary angiography, and cross-sectional views, similar to intravascular imaging. This provides interventional cardiologists with a familiar and useful visualization during PCI guidance. Thin-slab MIP guidance can be easily used on any workstation with commonly obtained thin-slice CT images.

### General CT evaluation of coronary lesions for slender PCI

Cardiac CT provides a thorough pre-procedural assessment, which is particularly beneficial for slender PCI planning. It allows for detailed evaluation of the coronary artery anatomy and lesion characteristics, categorized into anatomical assessment and lesion complexity analysis.

#### Anatomical assessment

Longitudinal and cross-sectional CT analysis provides precise measurements of lumen dimensions, including reference vessel diameter and lesion length, which are essential for appropriate stent sizing [17]. CT evaluation enables the accurate identification of optimal landing zones and the most effective fluoroscopic viewing angle. This is essential for improving fluoroscopic visualization by reducing vessel overlap and foreshortening. Consequently, it ensures a clear depiction of critical anatomy during the procedure [18]. Assessment of the coronary ostium position helps guiding catheter (GC) selection, while virtual reality-GC engagement simulation has recently been introduced [19]. This anatomical information significantly reduces the need for multiple contrast injections during the intervention, thereby minimizing both contrast agent volume and radiation exposure.

**Table 1:** Compatibility of device and technique according to size of guiding catheter

	Balloon	Stent	IVUS OCT/OFDI	Rotablator (Max bur size)	OAS	IVL (Max balloon size)	KBT	Two stent strategy
5 Fr	No limitation			1.25/1.5 mm	No limitation	3.5 mm	*Possible with limited devices and specialized techniques	*Possible with limited devices and specialized techniques
6 Fr				1.75 mm		4.0 mm	possible	possible
7 Fr				2.0 mm		4.0 mm	No limitation	No limitation

*Possible with limited devices and specialized techniques. See text for further detail

The inner lumen of guiding catheters and the outer diameters of balloons and stents vary among manufacturers; therefore, the aforementioned considerations cannot be universally guaranteed. Nevertheless, with careful device selection and the application of specialized techniques, interventions, such as KBT or a two-stent strategy, can be performed even using a 5-Fr guiding catheter. In all cases, comprehensive pre-procedural evaluation, including angiographic and/or CT imaging, is essential to ensure appropriate device selection and minimize the risk of device-related complications

*CT* computed tomography, *Fr* French, *IVL* intravascular lithotripsy, *IVUS* intravascular ultrasound, *KBT* kissing balloon technique, *OAS* orbital atherectomy system, *OCT* optical coherence tomography, *OFDI* optical frequency domain imaging

#### Lesion complexity analysis

CT evaluation offers a comprehensive characterization of plaque composition by distinguishing between fibrous, calcified, and lipid-rich plaques, which aids in selecting the appropriate device. Lesions with complex morphology, including heavily calcified lesions, significant bifurcation lesions, or total occlusion lesions, can be identified pre-procedurally, allowing for specific approaches or consideration of alternative treatment strategies. Assessment of vessel tortuosity and angulation helps predict device deliverability, which is particularly relevant for slender PCI systems that may have reduced navigability in tortuous anatomy. The ability to evaluate lesion complexity is crucial for slender PCI, as complex morphologies may challenge the capabilities of smaller devices.

Novel guide-extension catheters [20] and modification devices [21] compatible with 5 Fr GCs are emerging; however, the limited inner lumen of a GC and the potential for challenges with backup support remain key considerations in slender PCI [7]. This comprehensive anatomical and morphological information from cardiac CT facilitates informed decision-making in slender PCI.

### Bifurcation lesions

Bifurcation lesions represent one of the most challenging and popular anatomical subsets for slender PCI. In the management of such lesions, comprehensive pre-procedural CT assessment becomes crucial for optimal treatment planning and risk stratification [22]. The main limitation of slender PCI systems is their inability to accommodate multiple device insertions within a 5 Fr GC [7]. This restriction impacts even frequently used bifurcation PCI techniques, such as the kissing balloon technique and the jailed balloon technique, while the jailed side branch wire remains available. For these reasons, slender PCI is often avoided in bifurcation treatment. The operator’s concern regarding possible side branch occlusion leads to the use of a larger GC and the insertion of multiple guidewires. However, in numerous cases, the procedure can actually be completed with simple pre-dilatation and stent implantation. Hence, CT information may be the most useful tool to establish the correct strategy in advance and identify such cases suitable for slender PCI approaches.

CT-derived morphological parameters effectively predict the risk of side branch occlusion, with scoring systems showing excellent predictive performance across different bifurcation anatomies [23]. Critical risk factors identified through CT analysis include bifurcation angles, plaque distribution at the side branch ostium, calcium burden and its distribution, proximal vessel tortuosity, and the severity of baseline side branch stenosis [24, 25]. The amount of myocardial mass at risk (MMAR), which is easily quantifiable on CT [26], guides the treatment indication of jailed side branches, with > 10% MMAR generally considered a threshold for revascularization and preservation [27, 28]. While many side branches may appear to be at risk of compromise based on the risk scores, MMAR quantification frequently reveals that the territory supplied by these branches is < 10%. In such real-world settings, MMAR proves crucial for determining the appropriate indication for slender PCI by identifying side branches that do not genuinely require preservation. This comprehensive CT-based evaluation enables clinicians to determine whether complex bifurcation lesions are suitable for slender PCI approaches or require conventional larger-bore systems to accommodate dual-device strategies, thereby optimizing procedural success while minimizing complications.

### Calcified lesions

**Fig. 2:**
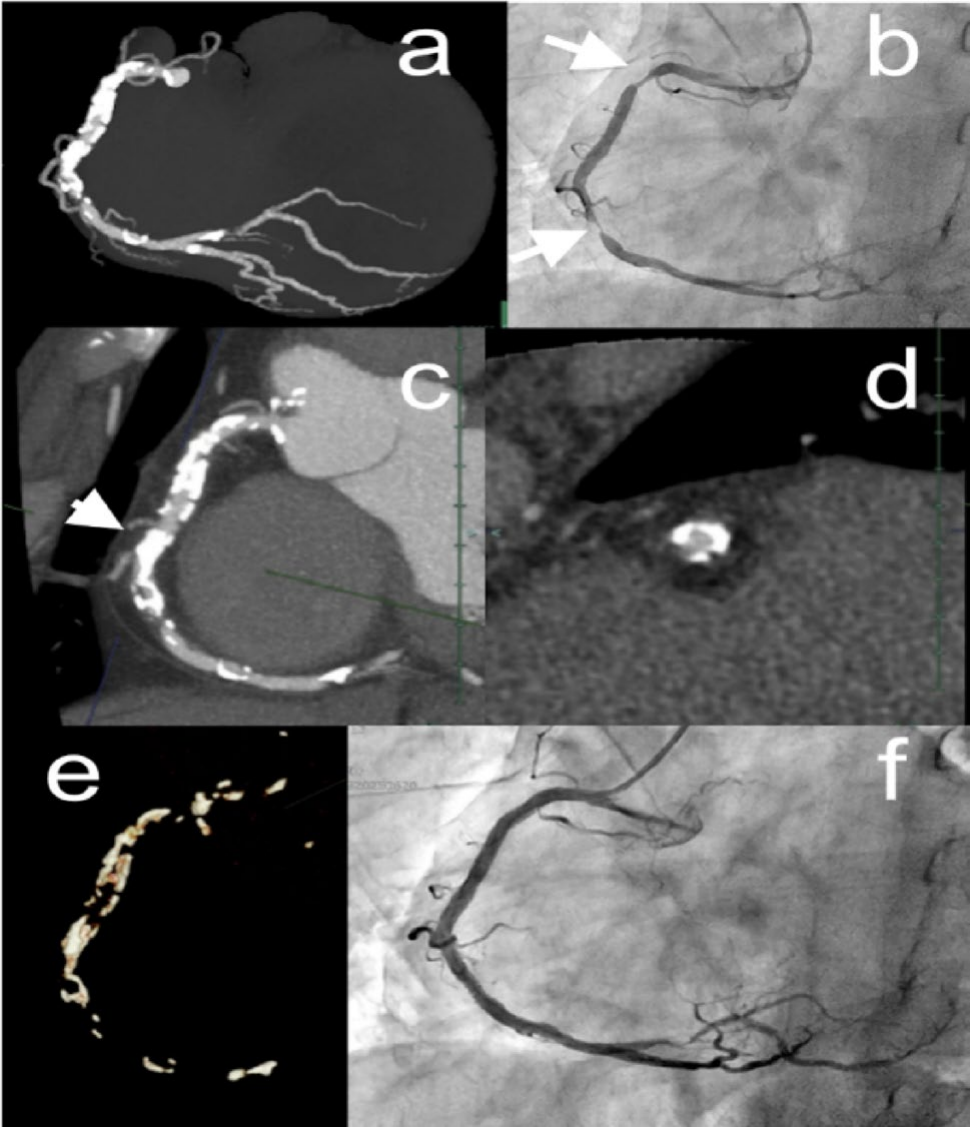
Pre-procedural cardiac CT assessment for slender PCI in a severely calcified Lesion. (**a**) A full-volume maximal intensity projection shows a calcified right coronary artery; however, precise evaluation of the luminal stenosis is difficult. (**b**) Coronary angiography shows proximal and mid lesions (arrows). (**c**) Thin-slab maximum intensity projection shows the calcified coronary artery. (**d**) The corresponding short-axial view at the most severely calcified lesion (arrowhead) reveals that the calcified plaque is non-circumferential and is divided into three segments. (**e**) 3D visualization of the calcified plaque shows that calcification is not circumferential. (**f**) Coronary angiography after the intervention shows the successful placement of two drug-eluting stents using a slender 5-Fr guiding catheter. *3D* three-dimensional, *CT* computed tomography, *Fr* French, *PCI* percutaneous coronary intervention

Heavily calcified coronary lesions present significant challenges for slender PCI systems and PCI in general. In practice, the available strategies for effective modification are limited. Thus, it is critically important to determine in advance whether these strategies are likely to be successful. Cardiac CT provides a comprehensive assessment of calcification distribution, extent, and characteristics that are crucial for procedural planning [29]. The evaluation should focus on the calcium arc, longitudinal length, calcium thickness, and the presence of calcified nodules or bifurcation involvement. Crucially, while intravascular imaging is the standard for detailed calcium assessment, it cannot be performed if the imaging catheter cannot cross the target lesion—a common scenario in heavily calcified vessels [30]. Cardiac CT overcomes this limitation by providing essential pre-procedural information regardless of lesion crossability, thus serving as a vital gatekeeper for complex PCI. Blooming artifacts complicate precise assessment; nevertheless, the calcium evaluation strongly correlates with the same metrics derived from intravascular imaging, particularly the calcium arc and length [31]. Calcium thickness can be estimated with the maximum CT density [32], while mean Hounsfield unit values > 637 suggest the need for rotational atherectomy [33]. The ABCD scoring system (Angle: ≥270°, Bifurcation lesions, Calcified mass, longitudinal Distance: ≥9 mm) has demonstrated utility in predicting the need for debulking devices with high sensitivity (76.0%) and specificity (82.5%) [34]. However, calcified nodules associated with poor outcomes after PCI have not been fully defined by CT findings [35]. Optimal visualization requires proper window settings (window level/width: 400–600/1,200–1,800) for cross-sectional analysis to evaluate calcium distribution patterns [12], as a full-moon appearance and circumferential calcification are indicators of poor outcomes in PCI [36, 37]. The 3D reconstruction of calcified plaque offers a strong advantage in visualizing the distribution, particularly circumferential calcification that is distributed obliquely to the long axis of the vessel. This is particularly important because short-axis views may not fully reveal a circumferential pattern, which can render lesions undilatable even with high-pressure balloon inflations. Severe calcification of stenotic lesions and segments proximal to the lesion can complicate delivery of the device. Previously, slender PCI systems were considered limited in accommodating certain modification devices, such as smaller burrs for rotational atherectomy or the technically demanding orbital atherectomy system [21]. Nonetheless, the recent introduction of intravascular lithotripsy up to 3.5 mm in size has significantly reduced these limitations. In turn, CT-derived calcium assessment enables operators to identify lesions suitable for slender PCI approaches, thereby preventing the unnecessary use of larger GCs and specialized devices (Fig. 2). Even when the coronary artery is highly calcified, pre-procedural calcium characterization is crucial for determining whether conventional systems with larger GCs and specialized devices are truly necessary for a safe and effective intervention.

### Chronic total occlusion (CTO) lesions

Treatment of CTO requires careful consideration of both procedural success rates and potential complications. Unfortunately, even in CTO expert registries, the reported success rates remain around 90%, accompanied by a relatively high incidence of complications [38, 39]. This inherent difficulty in CTO intervention underscores the critical need for meticulous pre-procedural planning.

In this context, cardiac CT evaluation plays a pivotal role in determining the feasibility and procedural strategy for all CTO interventions, including slender PCI. Precise characterization of the CTO entry point is essential, as it directly influences wiring success. CT provides essential information regarding key morphological features which are critical for antegrade wire navigation, such as proximal cap morphology (e.g., bluntness, taper, microchannels), the presence of side branches, and entry calcification.

This detailed CT-based assessment is vital for stratifying lesion complexity. Lesions exhibiting challenging features such as a blunt entry cap, long occlusion length, severe calcification, or significant tortuosity often require advanced techniques involving multiple device insertions or strong backup pushability of the GC. Consequently, operators frequently select a GC with a larger diameter to ensure device compatibility and support. However, this choice itself may contribute to procedural complications, thereby increasing the importance of accurate pre-procedural assessment.

To address this challenge, thorough pre-procedural cardiac CT evaluation is essential. CT-based assessment outperforms angiography in characterizing critical factors—specifically occlusion length, degree of calcification, and vessel tortuosity—which are directly linked to the need for large-bore systems. Adopting CT-based scoring systems for comprehensive lesion assessment is strongly recommended for all CTO cases to accurately gauge lesion difficulty [40–42]. In this context, the primary role of CT is to guide the strategy for slender PCI. Lesions classified by CT as having a tapered entry cap, short occlusion length, minimal calcification, and mild tortuosity are optimal candidates for a slender approach. Conversely, conventional systems with larger GCs are deemed truly necessary for a safe and effective intervention only when CT characterization indicates high complexity.

A retrograde approach remains an option also in slender PCI [43]. Nevertheless, its practical challenges coupled with the difficulty of precisely characterizing fine collaterals with CT necessitate careful integration with angiography. Indeed, compared with angiography-only guidance, CT guidance has been shown to improve procedural outcomes, achieving higher successful recanalization rates (84% vs. 94%, respectively, *p* = 0.003) and reduced coronary perforation rates [44]. Ultimately, CT evaluation is critical for appropriate case selection, as operators must recognize that complex CTO lesions may exceed the capabilities of slender PCI systems. The use of smaller GCs may theoretically increase procedural difficulty and prolong procedure, fluoroscopy time, and contrast agent volume due to reduced backup. Thus, meticulous pre-procedural CT planning is essential. By accurately identifying low-complexity lesions, CT ensures that slender PCI is selected only for cases where its use is efficient and safe.

Therefore, interventional cardiologists must aim to perform PCI procedures that are as minimally invasive and safe as contemporary surgical revascularization techniques. This philosophy necessitates a careful consideration of all factors that influence procedural safety, including the potential for complications associated with larger GCs—such as access site bleeding, coronary ostial injury, and increased contrast agent load. Hence, cardiologists must leverage pre-procedural CT to accurately identify optimal candidates for slender PCI and consistently prioritize the most minimally invasive approach.

### A proposed checklist for CT-guided slender PCI planning

**Fig. 3:**
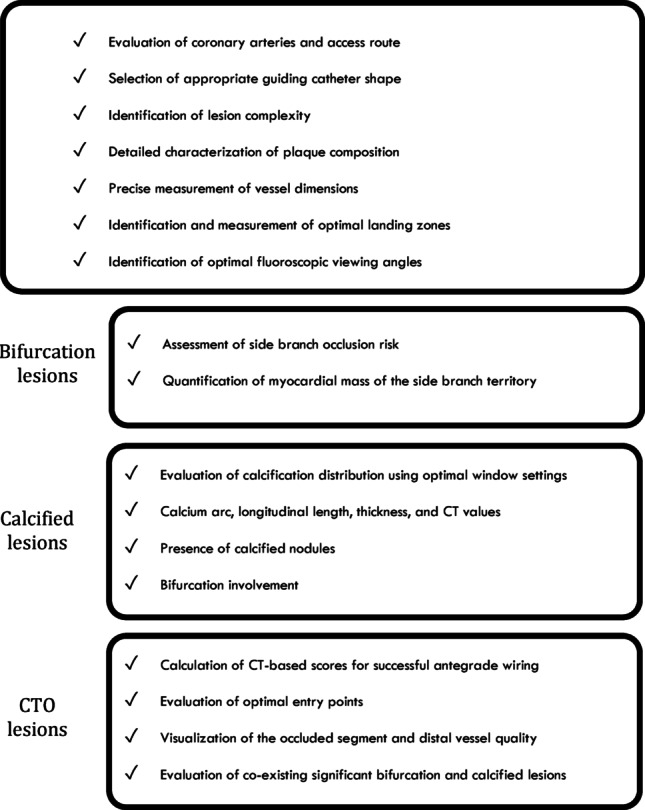
A pre-procedural checklist recommendation for cardiac CT-guided slender PCI planning. A proposed comprehensive checklist covering essential CT evaluation parameters for all coronary lesions, with specific additional considerations for bifurcation lesions, calcified lesions, and chronic total occlusion lesions. This systematic approach ensures thorough assessment of lesion complexity, plaque characteristics, and procedural feasibility to optimize patient selection and procedural planning for slender PCI strategies. *CT* computed tomography, *PCI* percutaneous coronary intervention

A pre-procedural checklist is proposed for the systematic application of the aforementioned comprehensive CT evaluation (Fig. 3). This checklist covers essential CT evaluation parameters for all coronary lesions, with specific additional considerations for bifurcation lesions, calcified lesions, and CTO lesions. This systematic approach ensures thorough assessment of lesion complexity, plaque characteristics, and procedural feasibility to optimize patient selection and procedural planning for slender PCI strategies. Furthermore, this CT-guided system is applicable to all PCI procedures, aiming to serve as a vital reference for providing safer and more effective treatment overall.

### Future directions and unmet needs

Several key areas require urgent investigation to establish the role of cardiac CT-guided slender PCI strategies. Most critically, prospective randomized trials comparing CT-guided versus angiography-guided slender PCI strategies are essential to provide definitive evidence for patient selection criteria and procedural outcomes. These studies should specifically address whether the theoretical advantages of CT guidance translate into measurable clinical benefits, such as reduced procedural complications, improved success rates, and enhanced cost-effectiveness.

The development of standardized CT acquisition protocols and reporting systems specifically tailored for slender PCI planning would enhance reproducibility and facilitate multi-center studies. Additionally, artificial intelligence-enhanced image analysis algorithms may be able to automate the assessment of lesion complexity and provide objective decision support for optimal equipment selection, thereby reducing operator-dependent variability. Long-term registry studies are warranted to evaluate the real-world effectiveness and cost-benefit ratio of routine CT guidance versus selective use in complex cases. Additionally, integrating CT-derived fractional flow reserve with anatomical assessment may improve the criteria for lesion selection, although this method needs validation in the context of slender PCI limitations.

## Conclusions

Cardiac CT is a crucial imaging modality that effectively addresses the limitations of slender PCI systems, such as restrictions on multiple device insertion and challenges with backup support. Its comprehensive 3D visualization allows for precise vessel measurements, accurate characterization of lesion complexity, and optimal equipment selection. The assessment is particularly important for complex lesions, such as bifurcation, heavily calcified, and CTO lesions, where CT offers detailed morphological information to guide treatment strategy. By incorporating cardiac CT using the proposed checklist into the workflow, interventionists can select the most suitable minimally invasive techniques from the beginning, thus improving procedural efficiency and optimizing resource utilization. Ultimately, cardiac CT optimizes the indications for slender PCI while enhancing the safety, efficacy, and success of the procedure.
